# Frequency‐dependent airway hyperresponsiveness in a mouse model of emphysema and allergic inflammation

**DOI:** 10.14814/phy2.13568

**Published:** 2018-01-25

**Authors:** Kentaro Tamura, Koichiro Matsumoto, Satoru Fukuyama, Keiko Kan‐o, Yumiko Ishii, Ken Tonai, Miyoko Tatsuta, Aimi Enokizu, Hiromasa Inoue, Yoichi Nakanishi

**Affiliations:** ^1^ Research Institute for Diseases of the Chest Graduate School of Medical Sciences Kyushu University Fukuoka Japan; ^2^ Department of Pulmonary Medicine Graduate School of Medical and Dental Sciences Kagoshima University Kagoshima Japan

**Keywords:** Airway hyperresponsiveness, asthma–COPD Overlap (ACO), dynamic lung compliance, frequency dependent, surfactant protein**‐**D (SP‐D)

## Abstract

Asthma and chronic obstructive pulmonary disease (COPD), chronic airway inflammatory diseases characterized by airflow limitation, have different etiologies and pathophysiologies. Asthma–COPD Overlap (ACO) has recently been used for patients with mixed asthma and COPD. The pathophysiological mechanisms of ACO have not been clearly understood due to the lack of an appropriate murine model. To investigate its pathophysiology, we examined a murine model by allergen challenge in surfactant protein**‐**D (SP‐D)‐deficient mice that spontaneously developed pulmonary emphysema. SP‐D‐deficient mice were sensitized and challenged by ovalbumin (OVA). Lungs and bronchoalveolar lavage fluid (BALF) were collected for analysis, and static lung compliance and airway hyperresponsiveness (AHR) were measured 48 h after the last OVA challenge. In SP‐D‐deficient, naïve, or OVA‐challenged mice, the mean linear intercept and static lung compliance were increased compared with wild‐type (WT) mice. There was no significant difference in goblet cell hyperplasia and the gene expression of Mucin 5AC (MUC5AC) between SP‐D‐deficient and WT OVA‐challenged mice. In SP‐D‐deficient OVA‐challenged mice, airway hyperresponsiveness was significantly enhanced despite the lower eosinophil count and the concentration of interleukin (IL)‐5 and IL‐13 in BALF compared with WT OVA‐challenged mice at 120 ventilations per minute. When mice were ventilated at a lower ventilation frequency of 100 ventilations per minute, elevated airway hyperresponsiveness in SP‐D‐deficient OVA‐challenged mice was diminished. This model of emphysematous change with allergic airway inflammation raises the possibility that frequency‐dependent airway hyperresponsiveness may be involved in the pathophysiology of ACO.

## Introduction

Asthma and chronic obstructive pulmonary disease (COPD) are recognized as chronic airway inflammatory diseases characterized by airflow limitation. They have been treated as independent diseases due to their differences in etiology and pathophysiology (Barnes 2006; GINA Science Committee, 2017; GOLD Science Committee, 2017). Increased infiltration of the lung with CD4^+^ lymphocytes and eosinophils has been reported in asthma, which induces bronchial mucosal edema, reversible airflow restriction, and airway hyperresponsiveness (GINA Science Committee, 2017). In contrast, in COPD, increased population of CD8^+^ lymphocytes and neutrophils in abnormal activation causes emphysematous changes and fibrosis of the bronchioles, resulting in irreversible airflow limitation (GOLD Science Committee, 2017).

However, it is sometimes difficult to distinguish clinically between asthma and COPD, especially in middle‐aged and elderly patients, and the term “asthma‐COPD Overlap” (ACO) has recently been used for patients with mixed asthma and COPD (Stankiewicz et al. 2002; Gibson and Simpson 2009; Matsumoto et al. 2015). ACO has been reported to show a high frequency of exacerbations, which leads to decreased quality of life (QOL) and poor prognosis (A Joint Project of GINA and GOLD, 2015). However, the pathophysiological mechanisms of ACO are not well understood due to the lack of an appropriate ACO animal model.

Surfactant protein‐D (SP‐D), a member of the collectin family secreted from type II alveolar epithelial cells and club cells, mainly functions as a biological defense lectin (Haczku 2008; Han and Mallampalli 2015). In transgenic mice lacking SP‐D, foamy macrophages that increase the production of H_2_O_2_ and matrix metalloproteinase (MMP) activity accumulate in the alveoli. Airspace enlargement is observed in SP‐D‐deficient mice by the postnatal age of 3 weeks (Wert et al. 2000), resulting in emphysematous lungs.

We previously reported airway hyperresponsiveness and inflammation using the ovalbumin (OVA)‐induced murine model of experimental asthma (Asai‐Tajiri et al. 2014). In this study, we established a murine model of ACO by OVA challenge in SP‐D‐deficient mice that spontaneously developed pulmonary emphysema and investigated airway hyperresponsiveness and inflammation. We also assessed whether ventilation rates have an effect on airway hyperresponsiveness in a murine model of ACO.

## Materials and Methods

### Animals

SP‐D^−/−^ mice with a C57BL/6 background were kindly provided by Dr. Jeffrey Whitsett (Cincinnati Children's Hospital Medical Center) and bred in the Kyushu University Animal Center. The colony of SP‐D‐deficient mice was reported to be maintained in filtered cages for more than 6 months, suggesting that SP‐D is not required for postnatal survival under vivarium conditions (Korfhagen et al. 1998). All procedures and protocols were approved by the Kyushu University Animal Care and Use Committee (reference number: A27‐056‐2).

### Sensitization and challenge

**Figure 1:**
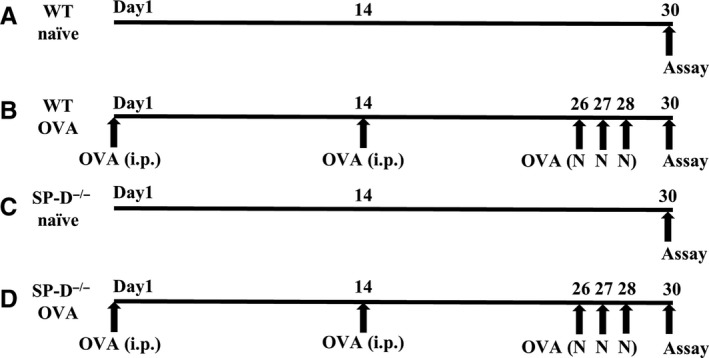
Experimental protocols. (A) Wild‐type (WT)/naïve mice were not sensitized or exposed to ovalbumin (OVA). (B) WT OVA‐challenged mice (WT/OVA) were sensitized on Days 1 and 14 and exposed to OVA on Days 26, 27, and 28. (C) SP‐D‐deficient (SP‐D^−/−^)/naïve mice were not sensitized or exposed to OVA. (D) SP‐D‐deficient OVA‐challenged mice (SP‐D^−/−^/OVA) were sensitized on Days 1 and 14 and exposed to OVA on Days 26, 27, and 28. Static compliance and airway hyperresponsiveness were measured, and lungs and BALF were collected on Day 30. i.p., intraperitoneal administration; N, nebulization.

Female mice (8‐week‐old) were sensitized with an intraperitoneal (i.p.) injection of 10 *μ*g of chicken OVA (Grade V, Sigma‐Aldrich, St. Louis, MO, USA) and 0.3 mg of Al(OH)_3_ (SERVA Electrophoresis, Heidelberg, Germany) on Days 1 and 14 and then 12‐week‐old mice were challenged with aerosolized 1% OVA for 30 min on Days 26, 27, and 28. Mice were assessed for airway hyperresponsiveness (AHR) followed by bronchoalveolar lavage (BAL) on Day 30, as previously described (Kibe et al. 2003). Experimental protocols are described in Figure 1.

### Histology

The lung was inflated and fixed by intratracheal instillation of 10% formalin. The transpulmonary pressure at which the lungs were inflated was 25 cm H_2_O static pressure (Ikeda et al. 2014). Tissue sections were stained with hematoxylin and eosin (HE) to assess the morphometric analysis to measure the alveolar size and stained with periodic acid‐Schiff alcian blue (PAS/AB) to assess goblet cell hyperplasia in the airways after antigen exposure.

### Morphometric analysis

Using a computer image analysis system, the cross‐sectional areas occupied by the wall and the luminal mucosa were quantified as ratios of the total cross‐sectional area (Hogg et al. 2004). Digitized video images of the entire lung fields were analyzed with a computerized color image analysis software system (Win Roof Version 6.1.1; Mitani Co., Fukui, Japan) (Hoshino et al. 2007).

### Static compliance

Mice were culled by injecting an overdose of sodium pentobarbital, and the trachea was cannulated and connected to a syringe linked to a pressure sensor (Differential Manometer DM‐280; As One Co., Osaka, Japan) via a three‐way connector. After opening the diaphragm, lungs were inflated in 100‐*μ*L increments every 10 sec to a maximum pressure of 25 cm of water and then deflated. Pressure–volume curves were generated for each animal (Wert et al. 2000).

### Determination of gene expression of MUC5AC

The expression of MUC5AC genes was analyzed using gel‐based reverse transcription–polymerase chain reaction (RT‐PCR) and real‐time RT‐PCR (Nakano et al. 2006; Ishii et al. 2016). Total RNA from the whole lung was isolated using the acid guanidinium thiocyanate‐phenol‐chloroform method and complementary deoxyribonucleic acid (cDNA) was generated using a PrimeScrip II first‐strand cDNA Synthesis Kit (Takara, Shiga, Japan). The *β*‐actin gene was used as an internal control. Primer sequences used in gel‐based RT‐PCR were as follows: mouse MUC5AC (*Muc5ac*) upstream region, 5′‐CAGCCGAGAGGAGGGTTTGATCT‐3′, and downstream, 5′‐AGTCTCTCTCCGCTCCTCTCAAT‐3′; mouse *β*‐actin (*Actb*) upstream, 5′‐TCCTGTGGCATCCATGAAACT‐3′, and downstream, 5′‐GAAGCACTTGCGGTGCACGAT‐3′. Primer sequences used in real‐time RT‐PCR were as follows: mouse MUC5AC (*Muc5ac*) upstream region, 5′‐CCTCTCAGAGGAATGTGACTCTGCGC‐3′, and downstream, 5′‐CCAGGCAGCCACACTTCTCAACCT‐3′; mouse *β*‐actin (*Actb*) upstream, 5′‐CACCTGGCAGAATTCCATCCG‐3′, and downstream, 5′‐CTCCCTGTTCACGGTCAGAG‐3′. Real‐time RT‐PCR reactions were performed using SYBR Premix Ex Taq II (Takara) on a Thermal Cycler Dice Real Time System II (Takara).

### Measurement of airway hyperresponsiveness (AHR)

AHR was measured according to our previously described protocol (Kibe et al. 2003). Briefly, mice were anesthetized with a mixture of ketamine and sodium pentobarbital i.p., and their tracheas were cannulated via tracheostomy. Mice were ventilated mechanically with a tidal volume of 0.3 mL and at a frequency of 120 breaths per minute or 100 breaths per minute. Rocuronium bromide, a muscle relaxant, was administered by i.p. injection. The airway opening pressure was measured with a differential pressure transducer and was recorded continuously. Stepwise increases in the acetylcholine dose (0.6–20 mg/mL) were given with an ultrasonic nebulizer (NE‐U07; OMRON Co., Kyoto, Japan) for 1 min. The data were expressed as the provocative concentration 200 (PC_200_), the concentration at which the airway pressure was 200% of its baseline value. The PC_200_ was calculated by log‐linear interpolation for individual animals as described previously (Kibe et al. 2003). Values of PC_200_ were expressed as log (PC_200 _× 100).

### Collection of bronchoalveolar lavage fluid (BALF) and measurement of cytokines and chemokines

Mice were given lethal doses of pentobarbital, and their lungs were gently lavaged once with 1.0 mL of 0.9% saline via the tracheal cannula. Total and differential bronchoalveolar lavage (BAL) cell counts were performed as described previously (Fukuyama et al. 2011). BALF was centrifuged at 250 *g* for 10 min, and the supernatants were stored at −80°C. Mouse interleukin (IL)‐5, IL‐13, IP‐10 (CXCL10), and KC (CXCL1) were measured using enzyme‐linked immunosorbent assay (ELISA) kits (R&D Systems, Inc., Minneapolis, MN, USA; detection limit 7.8–2000 pg/mL).

### Data analysis

Values were expressed as the means ± SEM. Interaction between SP‐D genotype and OVA challenge for each parameter was analyzed using two‐way ANOVA. Multiple comparison was conducted using Tukey–Kramer test. All statistical analyses were performed using GraphPad Prism6 (GraphPad Software, San Francisco, CA, USA). Results were considered statistically significant if *P *<* *0.05.

## Results

### SP‐D deficiency increases the pulmonary mean linear intercept and static lung compliance

**Figure 2:**
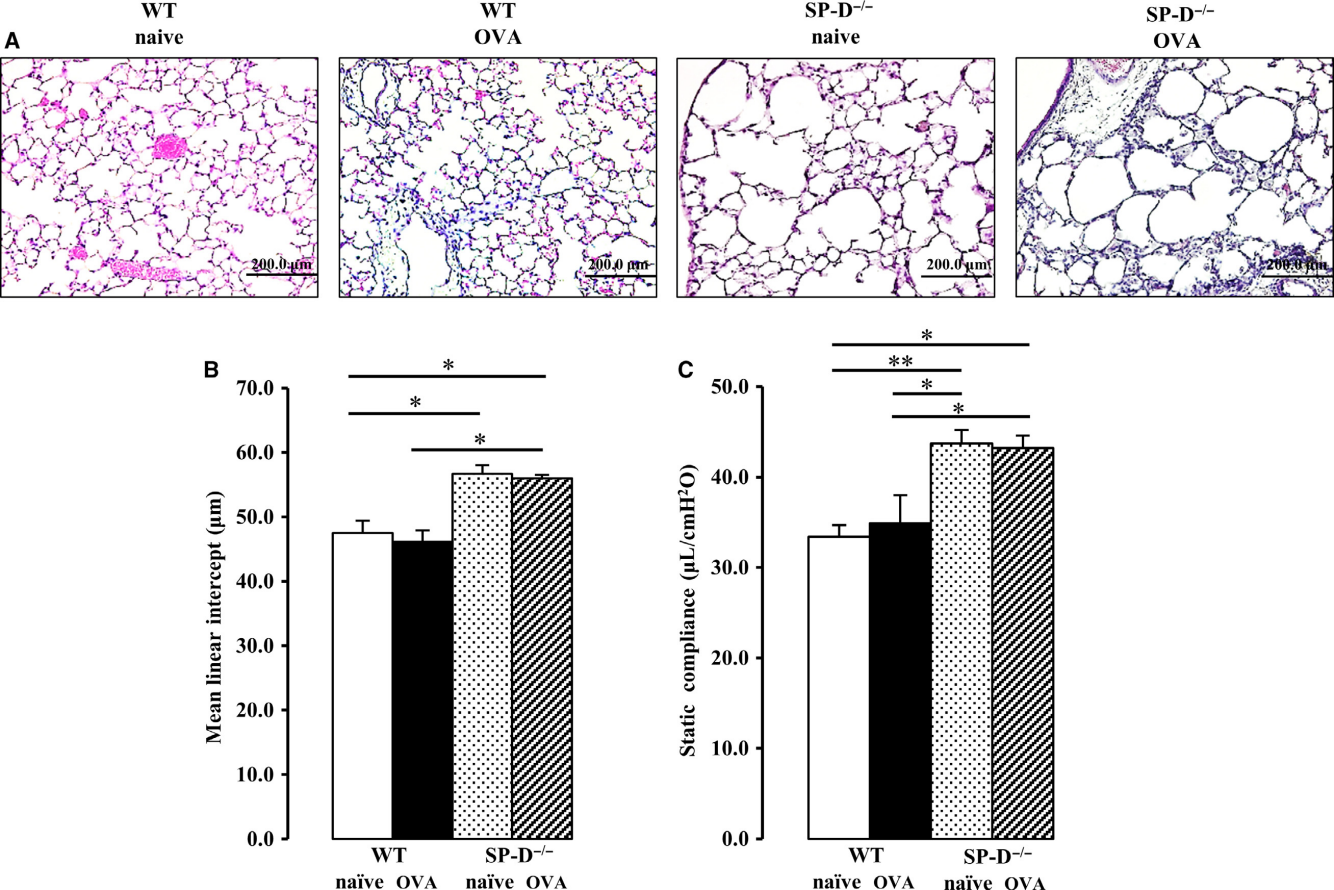
Effect of SP‐D deficiency on the mean linear intercept and static lung compliance. Static compliance was measured and the lungs were fixed 48 h after the last OVA challenge. (A) Representative images of hematoxylin and eosin (HE) staining (original magnification: ×200). (B) Emphysema assessed by the mean linear intercept. (C) Lung static compliance was measured. Data are mean ± SEM, *n* = 3–6 per group. *P <* *0.05, **P <* *0.01.

First, we evaluated the effect of SP‐D deficiency and OVA challenge on the pulmonary mean linear intercept and static lung compliance using SP‐D‐deficient mice. There was no interaction between SP‐D genotype and OVA challenge for the mean linear intercept and static lung compliance. The mean linear intercept was significantly increased in SP‐D‐deficient mice (Fig. 2A and B). However, OVA challenge had no effect on the mean linear intercept in either naïve or SP‐D‐deficient mice (Fig. 2A and B). Similar results were obtained when static lung compliance was analyzed. Static lung compliance was significantly increased in SP‐D‐deficient mice, and there was no significant difference in static lung compliance between naïve and OVA‐challenged mice (Fig. 2C).

### OVA sensitization and challenge, but not SP‐D deficiency, increases goblet cell hyperplasia

**Figure 3:**
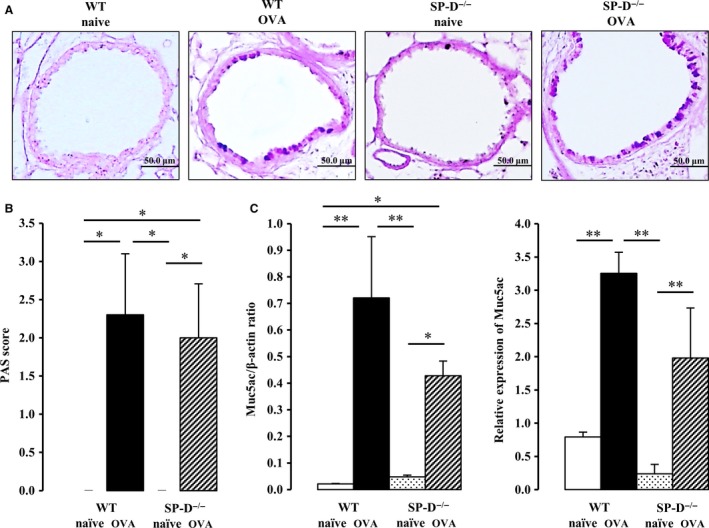
Effect of SP‐D deficiency and OVA exposure on goblet cell hyperplasia. Lungs were obtained 48 h after the last OVA challenge. (A) Representative images of periodic acid‐Schiff‐alcian blue (PAS/AB) to identify mucus‐containing cells (original magnification: ×200). (B) Semiquantitative analysis of the abundance of PAS‐positive cells. The numeric scores for the abundance of PAS‐positive, mucus‐containing cells in each airway were determined to be as follows: 0, <5% PAS‐positive cells; 1, 5–25%; 2, 25–50%; 3, 50–75%; 4, >75%. (C) Total RNA from the whole lung was isolated, and MUC5AC mRNA expression was estimated by performing gel‐based RT‐PCR (C, left panel). RT‐PCR products for *β*‐actin are shown for comparison. Gene expression of MUC5AC was determined by real‐time RT‐PCR and normalized to *β*‐actin (C, right panel). Data are mean ± SEM, *n* = 3–7 per group. *P <* *0.05, **P <* *0.01.

In asthmatic patients, airflow limitation is caused not only by airway contraction but also morphological changes, such as goblet cell hyperplasia in the airway epithelium (Tagaya and Tamaoki 2007). Next, we investigated the effect of SP‐D deficiency and OVA challenge on goblet cell hyperplasia in the bronchus. There was no interaction between SP‐D genotype and OVA challenge for AB/PAS‐positive cells. The number of AB/PAS‐positive cells in SP‐D‐deficient naïve mice was similar to that in WT naïve mice. After OVA challenge, AB/PAS‐positive cells were increased in WT and SP‐D‐deficient mice to the same extent (Fig. 3A). Goblet cell hyperplasia levels were not significantly different between WT and SP‐D‐deficient OVA‐challenged mice (Fig. 3B).

Similar results were obtained when we analyzed the MUC5AC gene expression of the lung, a molecular marker of goblet cell hyperplasia. There was no interaction between SP‐D genotype and OVA challenge for MUC5AC mRNA expression. There was also no significant difference in the levels of MUC5AC gene expressed by WT and SP‐D‐deficient naïve mice. However, MUC5AC gene expression was markedly induced by OVA challenge in WT and SP‐D‐deficient mice (Fig. 3C).

### Airway hyperresponsiveness, but not eosinophilic inflammation in the lungs, is elevated at high ventilation rates in a murine model of ACO

**Figure 4:**
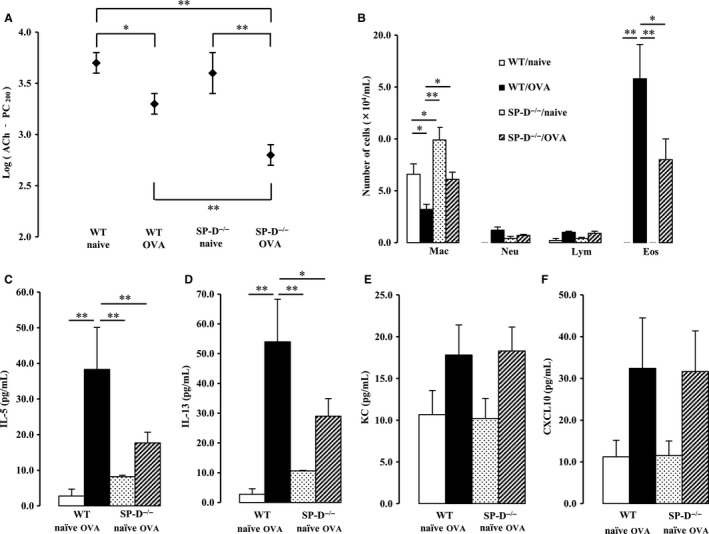
Effect of SP‐D deficiency and OVA exposure on airway hyperresponsiveness at high ventilation rates. Mice were ventilated at 120 ventilations per minute 48 h after the last OVA challenge. (A) Airway hyperresponsiveness was determined by the acetylcholine‐dependent change in airway pressure, and the provocative concentration at which the airway pressure was 200% of its baseline value (PC_200_)‐Ach was calculated. (B) Cell counts in bronchoalveolar lavage (BAL) fluid were performed. Mac, macrophage; Neu, neutrophil; Lym, lymphocyte; Eos, eosinophil. (C–F) Proinflammatory cytokine and chemokine levels in BAL fluid were measured. Data are the mean ± SEM, *n* = 7–13 per group. *P <* *0.05, **P <* *0.01.

Next, we examined the effect of SP‐D deficiency on airway hyperresponsiveness when mice were ventilated at a high rate (120 per minute). There was a significant interaction between SP‐D genotype and OVA challenge for airway hyperresponsiveness (*P *<* *0.05). Airway hyperresponsiveness was increased in WT OVA‐challenged mice, as we previously reported (Asai‐Tajiri et al. 2014). Furthermore, and interestingly, airway hyperresponsiveness was elevated in SP‐D‐deficient OVA‐challenged mice compared with WT OVA‐challenged mice (Fig. 4A).

However, unexpected results were obtained in the analysis of cell components, cytokines and chemokines in BALF. A significant interaction was seen between SP‐D genotype and OVA challenge for the number of eosinophils in BALF (*P *<* *0.05). A significant increase in the number of eosinophils in BALF was observed in OVA‐challenged group; however, the degree of eosinophilia was significantly lower in SP‐D‐deficient OVA‐challenged mice than in WT OVA‐challenged mice (Fig. 4B). In addition, SP‐D‐deficient naïve and OVA‐challenged mice showed an increase in the number of macrophages in BALF, compared with WT mice (Fig. 4B).

The production of cytokines and chemokines in BALF was measured. There were significant interactions between SP‐D genotype and OVA challenge for IL‐5 and IL‐13, T‐helper (Th) 2 cytokines (*P *<* *0.01, *P *<* *0.01, respectively). IL‐5 and IL‐13 were significantly induced in the OVA‐challenged group; however, the degree of increase in IL‐5 and IL‐13 was again significantly lower in SP‐D‐deficient OVA‐challenged mice than in WT OVA‐challenged mice (Fig. 4C and D). Increases in the production of KC and CXCL10 were observed in the OVA‐challenged group; however, there was no significant difference in production levels between WT and SP‐D‐deficient OVA‐challenged mice (Fig. 4E and 4F).

### Increased airway hyperresponsiveness is attenuated at low ventilation rates in a murine model of ACO

**Figure 5:**
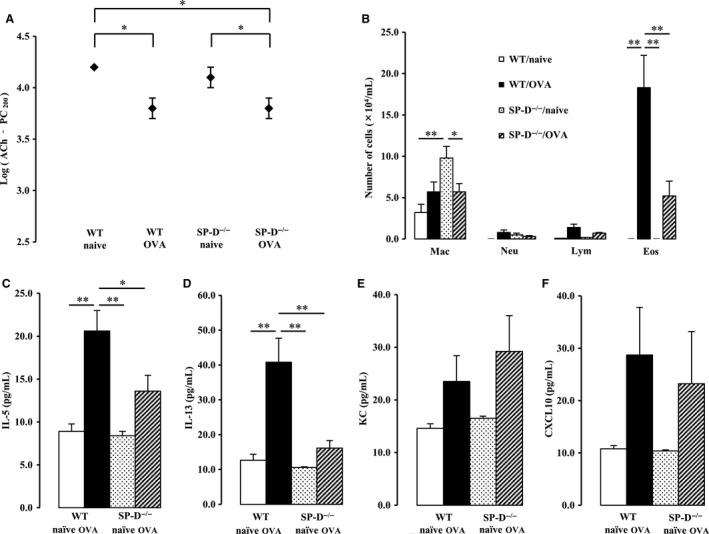
Effect of SP‐D deficiency and OVA exposure on airway hyperresponsiveness at low ventilation rates. Mice were ventilated at 100 ventilations per minute 48 h after the last OVA challenge. (A) Airway hyperresponsiveness was determined by the acetylcholine‐dependent change in the airway pressure, and the provocative concentration at which the airway pressure was 200% of its baseline value (PC_200_)‐Ach was calculated. (B) Cell counts in bronchoalveolar lavage (BAL) fluid were performed. Mac, macrophage; Neu, neutrophil; Lym, lymphocyte; Eos, eosinophil. (C–F) Proinflammatory cytokine and chemokine levels in BAL fluid were measured. Data are the mean ± SEM, *n* = 5–11 per group. *P <* *0.05, **P <* *0.01.

To evaluate the effect of ventilation rates on airway responsiveness, mice were ventilated at a low rate (100 per minute). Increased airway hyperresponsiveness at high rates in SP‐D‐deficient OVA‐challenged mice was attenuated at the same levels of WT OVA‐challenged mice (Fig. 5A). There was no interaction between SP‐D genotype and OVA challenge for airway hyperresponsiveness.

Again, a significant increase in the number of eosinophils in BALF was observed in the OVA‐challenged group; however, the degree of eosinophilia was significantly lower in SP‐D‐deficient OVA‐challenged mice than in WT OVA‐challenged mice (Fig. 5B). In addition, SP‐D‐deficient naïve mice showed an increase in the number of macrophages in BALF compared with WT naïve mice (Fig. 5B). The patterns of cytokines and chemokines released at low rates were similar to those seen at high ventilation rates (Fig. 5C–F).

## Discussion

To investigate the pathophysiological mechanisms of ACO, we established and examined a murine model by allergen (OVA) challenge in SP‐D‐deficient mice that spontaneously developed pulmonary emphysema. The mean linear intercept and static lung compliance were increased in SP‐D‐deficient mice compared with WT mice. Goblet cell hyperplasia and the gene expression of MUC5AC were induced only in OVA‐challenged mice. Airway hyperresponsiveness was augmented at a high ventilation rate (120 per minute) in a murine model of ACO, however, lower eosinophil count and the concentration of IL‐5 and IL‐13 in BALF were observed. Finally, elevated airway hyperresponsiveness in SP‐D‐deficient OVA‐challenged mice was diminished at a lower ventilation frequency (100 per minute).

The increase in mean linear intercept and the static lung compliance in 12‐week‐old SP‐D‐deficient mice may indicate that emphysematous changes developed. It is known that patients with asthma show phenotypes of airway remodeling, such as mucus secretory cells proliferation, subepithelial fibrosis, and increases in airway smooth muscle. In contrast, inflammation with mainly CD8^+^ lymphocytes and neutrophils causes pulmonary emphysema and fibrosis of the bronchioles in COPD. However, in our studies, there were no significant differences in goblet cell hyperplasia and MUC5AC mRNA expression between OVA‐challenged SP‐D‐deficient and WT mice, and emphysematous changes in the lungs were exclusively observed in SP‐D‐deficient mice.

The time constant is the product of static lung compliance and airway resistance. Emphysematous changes in the alveolar region increase lung compliance, while allergic airway inflammation increases airway resistance, leading to the rise of a time constant and substantial effect on the frequency‐dependent dynamic of compliance in the lungs (de la Hoz et al. 2000). In this study, airway hyperresponsiveness was augmented in SP‐D‐deficient OVA‐challenged mice despite milder eosinophilic inflammation compared with WT mice, and so we investigated whether similar phenomena would be observed if ventilation rates were reduced. At a low ventilation rate, the augmented airway hyperresponsiveness seen at the high ventilation rate was diminished, although eosinophilic inflammation was unchanged (Kirby et al. 1987; Walker et al. 1991). Rising of the time constant may be related to an increase in regional inhomogeneity which is derived not only from allergic inflammation in the small airways but also from heterogeneous emphysematous changes in the alveoli. The positive effect of this mechanism on airway responsiveness may still be relevant in less allergic inflammation in SP‐D‐deficient mice than that in WT mice. Because dynamic compliance is frequency dependent, lower ventilatory frequency would lessen or nullify the impact of increased inhomogeneity on airflow obstruction. These results indicate that the coexistence of allergic airway inflammation and emphysematous changes causes excessive airway obstruction at high ventilation rates and may augment airway hyperresponsiveness. The frequency‐dependent airway hyperresponsiveness in this study may be one of the ACO‐specific pathophysiological mechanisms by which we can explain an important clinical problem with lower QOL in patients with ACO. Subjects with ACO have been reported to show more respiratory symptoms, including cough, wheezing, and dyspnea, than those with asthma or COPD alone (Menezes et al. 2014). Dyspnea is enhanced with an increase in respiratory rates during exercise. Hence, dyspnea in ACO would be mainly attributed to the increased time constant produced by asthmatic airway and emphysema in parenchyma.

In this model, SP‐D‐deficient mice that spontaneously developed emphysematous changes were sensitized and challenged with OVA to establish an experimental asthma model. Interestingly, there were significantly lower numbers of eosinophils and production of Th2 cytokines in the BALF of SP‐D‐deficient mice challenged with OVA compared with WT mice challenged with OVA. SP‐D is a hydrophilic glycoprotein that has been reported to bind to various bacteria, viruses, allergens, and apoptotic cells via a C‐type carbohydrate‐recognition domain (CRD), function as an opsonin and regulate the production of cytokines and inflammatory mediators by acting directly on immune cells (LeVine and Whitsett 2001; Wright 2005). SP‐D promotes acquired immunity via enhancing antigen uptake and presentation by dendritic cells (Brinker et al. 2001; Wright 2005), suggesting that deficiency of SP‐D would reduce allergic inflammation. Additionally, a recent study reported that SP‐D is responsible for IL‐5‐induced eosinophilic inflammation in *Cryptococcus*‐infected mice (Holmer et al. 2014). Although the model is different to our allergic model, it partly explains decreased IL‐5 production and airway eosinophilia in SP‐D deficiency. However, a previous study showed that the number of eosinophilia and the production of Th2 cytokines increased in SP‐D‐deficient mice compared with WT mice after OVA challenge (Schaub et al. 2004), while another study showed no difference in eosinophilia between SP‐D‐deficient and WT mice (Fakih et al. 2015). The different protocols of OVA sensitization/challenge may be associated with the different results among these studies.

Emphysematous changes in the lungs can be developed in experimental models of COPD using elastase or genetic modification of SP‐D. In those models, unlike the model of COPD by chronic exposure to cigarette smoke with harmful particles and gaseous substances, chronic airway inflammation is not induced (Gan et al. 2004). However, ACO is also common in the elderly (de Marco et al. 2013), and substantial proportions of them are never or former smokers. Nagai et al. (2006) reported that the number of neutrophils and macrophages in BALF did not significantly increase in former smokers who had emphysematous changes and had stopped smoking for more than a year compared with never smokers. Hence, the model in this study is considered to be a disease model of ACO when the effect of the overlap between emphysema and asthma on ventilatory mechanics is investigated. Interestingly, there is a report that the concentration of SP‐D in BALF was lower in middle‐aged and elderly current smokers who had emphysematous changes than in never smokers (Betsuyaku et al. 2004).

In summary, we demonstrated that SP‐D‐deficient mice developed emphysema and that airway hyperresponsiveness was enhanced at high ventilation rates in SP‐D‐deficient OVA‐challenged mice despite milder eosinophilic inflammation in the lungs compared with WT OVA‐challenged mice. The increased airway hyperresponsiveness was diminished if mice were ventilated at low ventilation rates, raising the possibility that frequency‐dependent airway hyperresponsiveness may be involved in the pathophysiology of ACO.

## Conflict of interest

No conflicts of interest, financial or otherwise, are declared by the author(s).
